# fitgrid: A Python package for multi-channel event-related time series regression modeling

**DOI:** 10.21105/joss.03293

**Published:** 2021-07-19

**Authors:** Thomas P. Urbach, Andrey S. Portnoy

**Affiliations:** 1 Department of Cognitive Science, University of California, San Diego

## Abstract

Electrical brain activity related to external stimulation and internal mental events can be measured at the scalp as tiny time-varying electric potential waveforms (electroencephalogram; EEG), typically a few tens of microvolts peak to peak (Berger, 1930). Even tinier brain responses, too small to be seen by naked eye in the EEG, can be detected by repeating the stimulation, aligning the EEG recordings to the triggering event and averaging them at each time point (Dawson, 1951, 1954). Under assumptions that the brain response (signal) is the same in each recording and the ongoing background EEG (noise) varies randomly, averaging improves the estimate of the “true” brain response at each time point as the random variation cancels. The average event-related brain potential (ERP) and its counterpart for event-related magnetic fields (ERFs) are cornerstones of experimental brain research in human sensation, perception, and cognition (Luck & Kappenman, 2013).

Smith and Kutas pointed out that the average ERP at each time *t* is mathematically identical to the estimated constant ${\hat{\beta }}_{0}(t)$ for the regression model *y*(*t*) = *β*_0_(*t*) + *ε*(*t*), fit by minimizing squared error (Smith & Kutas, 2015a). The average ERP can be viewed as a time series of model parameter estimates. Generalizing to more complex models such as multiple regression *y* = *β*_0_ + *β*_1_*X*_1_ + … + β*_p_X_p_* + *ε*, likewise produces time series of estimates for the constant and each regressor coefficient, the ${\hat{\beta }}_{0}(t),{\hat{\beta }}_{1}(t),\ldots ,{\hat{\beta }}_{p}(t)$ dubbed regression ERP (rERP) waveforms (see Smith & Kutas, 2015a, 2015b for discussion of related approaches). This holds for straight-line fits (“slope” rERPs) as well as models of curvilinear relationships such as spline regression (Smith & Kutas, 2015b). Besides the estimated coefficient rERPs, the approach also produces time series for all the basic and derived quantities of the fitted model: coefficient standard errors, residuals, likelihood, Akaike information criterion (AIC), and so forth. With the shift from averaging to regression modeling, however, comes a new problem: fitting, diagnosing, comparing, evaluating and interpreting large numbers of regression models.

## Statement of need

Interpreting recordings of brain responses and drawing inferences from patterns of systematic variation is based on statistical comparison and evaluation of candidate models. Whereas fitting a regression model is straightforward on current scientific computing platforms, informative modeling, by contrast, is a laborious process that iterates cycles of data quality control, fitting, data diagnosis, model evaluation, comparison, selection, and interpretation with numerous decision points that require thought and judgment.

Modeling digitized multichannel EEG data as regression ERPs at each time point and data channel multiplies the iterative cycles in a combinatorial explosion of times × channels × models × comparisons. For instance, at a digital sampling rate of 250 samples per second, in 3 seconds of 32-channel EEG data there are 24,000 data sets (= 3 × 250 × 32). To fit a set of three candidate models requires 72,000 separate model fits, where the size of each data set might range anywhere from a few dozens of observations for a single subject to tens of thousands of observations for a large scale experiment.

The combinatorial explosion of model fits is unavoidable; fitgrid contains it by gathering the rich fit objects in a regular Time x Channel grid that provides users with approachable access to corresponding grids of the fit objects’ attributes. There are various Python implementations that use matrix operations operations to efficiently estimate ordinary linear regression coefficients *en masse* for N-D arrays of 1-D vectors such as numpy.linalg.lstsq (Harris et al., 2020) or sklearn.linear_model.LinearRegression (Pedregosa et al., 2011). By capturing the statsmodels and pymer4.Lmer model fit objects on tidy grids, fitgrid provides users with equally approachable access to the estimated coefficients and, crucially, the other quantities baked into the fit result objects such as coefficient standard errors, residuals, likelihood, and goodness of fit measures required for evaluating, comparing, and interpreting models and, ultimately, drawing inferences about the estimated coefficients.

The rERP approach is attracting growing attention in the field but to date the open-source EEG and magnetoencephalography (MEG) data analysis landscape has been dominated by toolboxes written for MATLAB such as EEGLAB (Delorme & Makeig, 2004), FieldTrip (Oostenveld et al., 2011), and Brainstorm (Tadel et al., 2011), and this holds for rERP modeling (e.g., Ehinger & Dimigen (2019)). Like open-source scientific computing generally, Python and R have been gaining traction for EEG and MEG analysis, as in MNE-Python Gramfort et al. (2013), and for regression ERPs in R Tremblay & Newman (2015). Nevertheless, widely accessible implementations for rERP modeling in Python remain limited. Development of N. J. Smith’s promising rERPy Python package for ERP and rERP analysis appears to have halted in Python 2, which reached its end of life in January 2020. MNE-Python implements a linear_regression function for computing rERP coefficients on continuous data as described in Smith & Kutas (2015b) but not time series of OLS or mixed-effects model fits. fitgrid is intended to fill this gap in the Python ecosystem.

## fitgrid

fitgrid makes the rERP modeling described in Smith & Kutas (2015a) accessible to researchers with a working knowledge of scripted data analysis in Python and the symbolic formulae such as ~ 1 + a + b + a : b and ~ 1 + a * b + (a∣s) + (a∣i) currently in wide use to specify ordinary and mixed-effects models in Python and R (patsy Smith, 2011-2015; lme4::lmer Bates et al., 2015; lm R Core Team, 2020).

The fitgrid user interface launches what are routinely hundreds to tens of thousands of model fits with one line of code (computed in parallel if supported by hardware). The fit results across times and channels are available on demand with the same syntax used to access results in a single fit object and the results are returned as tidy indexed pandas.DataFrames (McKinney 2010) for further analysis, visualization, and interpretation.

fitgrid provides routines for generating simulated data and downloading sample EEG data from a public Zenodo companion archive (Urbach, 2020). The documentation includes executable Python vignettes that illustrate their use. While the origins of fitgrid are in EEG data analysis, fitgrid can also be used with other neuroimaging data such as MEG and more generally with synchronized sensor array time-series data from other domains for which event-related regression modeling is appropriate. fitgrid enables researchers to conduct this type of computationally-intensive modeling flexibly efficiently informatively and reproducibly with familiar scientific computing tools and minimal programming. These features make fitgrid well-suited for general use in exploratory data analysis (EDA; e.g., Urbach et al. (2020) and Troyer et al. (2020)).

## Documentation

The fitgrid documentation is available online: https://kutaslab.github.io/fitgrid.

- Installation gives instructions, options, and examples for installing and fitgrid in conda virtual environments. Installation with pip is not supported because of the numerous R dependencies.
  Installation of the stable release into a fresh conda virtual environment along with other compatible packages, such as Jupyter or JupyterLab for running Example Gallery notebooks, is recommended. To install fitgrid in a conda environment named fg_env with the additional package dependencies downloaded primarily from the conda-forge channel run this:

$ conda create --name fg_env \
    fitgrid jupyter \
    -c kutaslab -c ejolly -c conda-forge \
    --strict-channel-priority

To install fitgrid with dependencies downloaded primarily from the Anaconda default channels (main, r), run this:

$ conda create --name fg_env \
    fitgrid jupyter \
    -c kutaslab -c ejolly -c defaults -c conda-forge

- Quick Start gives an overview of the fitgrid workflow with notes, matplotlib figures (Hunter, 2007), and downloadable and executable code.
- The User Guide provides information about usage and specific topics including how the OLS models are fit in Python statsmodels (Seabold & Perktold, 2010) and the LMER models are fit in R (lme4::lmer, lmerTest Kuznetsova et al., 2017) via pymer4 (Jolly, 2018) and rpy2 (Gautier, 2021).
- The API is a complete listing of fitgrid classes, methods, attributes, and functions auto-generated with numpy-style docstrings and links to the source code generated by sphinx-apidoc (Brandi & others, 2007-2021).
- The Examples Gallery contains annotated fitgrid vignettes with simulated data, experimental EEG recordings, and NOAA tide and atmospheric observations. The examples illustrate how to prepare data for modeling, fit ordinary least squares and linear mixed-effects models, summarize, and visualize the results. All the examples can be downloaded as executable Python scripts or Jupyter notebooks thanks to Sphinx-Gallery.
- The Bibliography includes references to relevant experimental and technical literature.
- The Contributing section encourages users and developers to post field reports and ideas large and small for improving fitgrid in the GitHub Issues in accordance with the Code of Conduct. It also gives an overview for developers and instructions for configuring a conda development environment and installing fitgrid from source for the purpose of modifying the code or documentation.

### Source, Continuous Integration, Packaging and Deployment

fitgrid has been developed and tested locally on 48-core x86_64 CentOS 7 and 144-core x86_64 Ubuntu 20.04 servers with Intel Xeon CPUs.

The source code is hosted in the public GitHub repository github.com/kutaslab/fitgrid.

The latest stable release of fitgrid and the bleeding edge pre-release development version are packaged for Python 3.7, and 3.8 on x86_64 linux and Intel OSX platforms and distributed on anaconda.org/kutaslab.

The continuous integration and deployment (CID) is implemented in a single-pass GitHub Action workflow, figrid-cid.yml. The continuous integration builds and installs the conda package in a conda virtual environment, and runs pytests and generates the sphinx documentation with the conda package as installed. The deploy phase automatically uploads the conda packages and documentation for development version pre-releases and stable releases and synchronizes the stable release source code across the GitHub repository, the conda packages at anaconda.org/kutaslab/fitgrid, the online sphinx documentation kutaslab/github.io/fitgrid, and the Zenodo source code archive at 10.5281/zenodo.3581496.

The continuous integration workflow for the latest stable release on the main code branch runs nightly on GitHub Action hosted runners. Pre-release packages also pass the CI conda build, install, pytest, and document generation phases before deployment to anaconda.org/kutaslab/fitgrid/label/pre-release. Python 3.7 and 3.8 64-bit Windows conda packages are also distributed but not routinely tested.

### Implementation

The core function of fitgrid is to apply a linear regression or a linear mixed effects model to datasets encapsulated in an input Epochs object. The output is a FitGrid which maintains a 2D grid of fit objects, each representing the results of fitting the model on the dataset associated with the grid point.

The FitGrid object exposes the same attributes and methods that the individual fit objects provide. Attribute accesses and method calls are broadcast to each cell in the grid with results returned in a form that mirrors the shape of the original grid, eliminating the need for explicit iteration over the elements. NumPy array slicing syntax is supported for indexing on the grid dimensions (time and channels when applied to EEG data). This functionality is achieved by implementing some of Python’s special methods like __getitem__, __getattr__, __ call__, __dir__ in the FitGrid class.

As a result, the cognitive load on the researcher is lightened since the familiar interfaces of a fit object (used to examine model fit characteristics) and of a 2D NumPy array (used to index on the space and time dimensions) are combined in a single entity.

When multiple model formulations are used, the resulting FitGrid objects can be used to compare goodness of fit measures and carry out mass model comparison and selection. This is one of the main applications enabled by the framework described in this section.

### Limitations

In addition to straight-line fits, the fitgrid framework can fit OLS models of curvilinear relations between predictors and EEG with model formulas because patsy supports column variable transformation by arbitrary Python code. Polynomial regression ERPs for U-shaped relations can be computed with, for example, x + pow(x, 2) if this seems like a good idea. If spline regression as described in Smith & Kutas (2015b) seems like a better idea, patsy also provides built-in functions for generating families of spline bases, although the researcher is responsible for ensuring that the data epochs are appropriately mapped to the spline regression variables which may require additional programming. Smith & Kutas (2015b) also generalizes rERP estimation from iteratively fitting fixed-length epochs of length *L* at each time point to fitting continuous data with a single model. For a design with *P* predictor variables, this conceptually elegant approach unstacks the *L* times (rows) of the epoch into L × P predictor variables (columns) and codes the observation row values as zeros or non-zeros according to the value of the predictor at the time. The coefficients estimated by a single OLS fit are identical to segmenting the data and fitting models with the *P* predictors iteratively at each of the *L* time points. In principle fitgrid can ingest and fit the continuous data prepared for the wide *L* × *P* design matrix as a corner case of single-sample epochs but it is not a natural act and at cross-purposes in some respects. In fitgrid, models are fit separately at each time and channel in order to track the time course of all the fit attributes, not just the estimated regression coefficients. For fitting the wide L × P models to continuous data, implementations specifically designed for that approach such as the rERPy package or the implementation in MNE-Python may be a better choice.

## Figures and Tables

**Figure 1:**
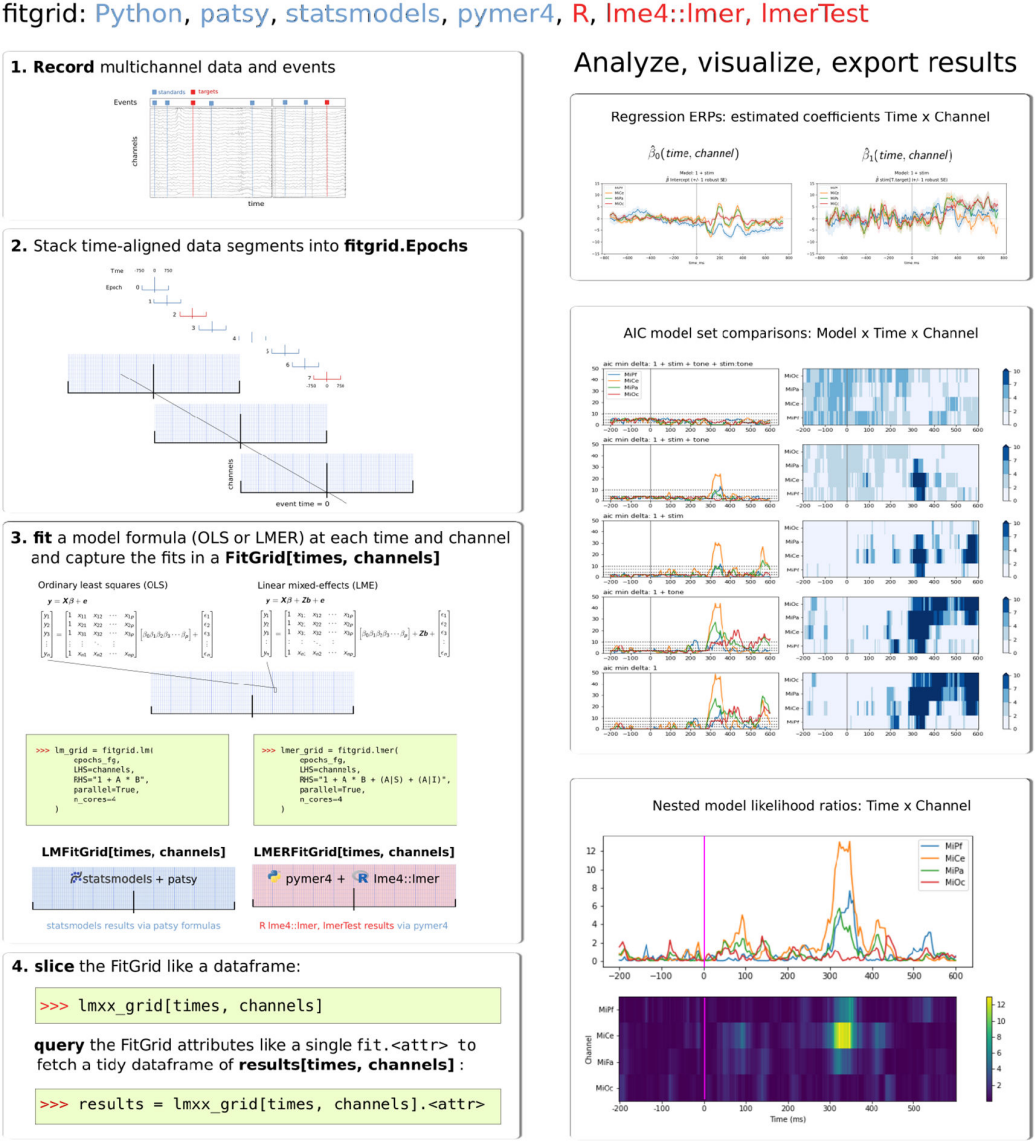
fitgrid Overview. The left column shows the four basic steps to set up and compute regression models for single-trial data epochs with fitgrid. The right column shows example visualizations of the modeling results.

**Figure 2:**
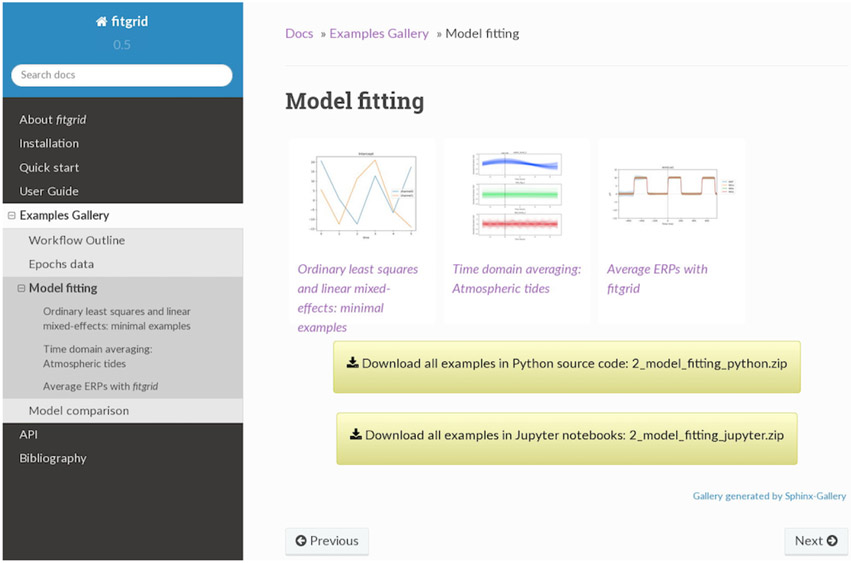
Examples such as those shown here can be downloaded as Python scripts or Jupyter notebooks and run on the user’s local machine after installing fitgrid.
